# Mutations in PpAGO3 Lead to Enhanced Virulence of *Phytophthora parasitica* by Activation of 25–26 nt sRNA-Associated Effector Genes

**DOI:** 10.3389/fmicb.2022.856106

**Published:** 2022-03-24

**Authors:** Junjie Xu, Yilin Li, Jinbu Jia, Wenjing Xiong, Chengcheng Zhong, Guiyan Huang, Xiuhong Gou, Yuling Meng, Weixing Shan

**Affiliations:** ^1^State Key Laboratory of Crop Stress Biology for Arid Areas and College of Plant Protection, Northwest A&F University, Yangling, China; ^2^State Key Laboratory of Crop Stress Biology for Arid Areas and College of Agronomy, Northwest A&F University, Yangling, China; ^3^State Key Laboratory of Crop Stress Biology for Arid Areas and College of Life Sciences, Northwest A&F University, Yangling, China

**Keywords:** *Phytophthora parasitica*, AGO3, small RNA, RXLR effector, RGG domain

## Abstract

Oomycetes represent a unique group of plant pathogens that are destructive to a wide range of crops and natural ecosystems. *Phytophthora* species possess active small RNA (sRNA) silencing pathways, but little is known about the biological roles of sRNAs and associated factors in pathogenicity. Here we show that an *AGO* gene, *PpAGO3*, plays a major role in the regulation of effector genes hence the pathogenicity of *Phytophthora parasitica*. *PpAGO3* was unique among five predicted *AGO* genes in *P. parasitica*, showing strong mycelium stage-specific expression. Using the CRISPR-Cas9 technology, we generated *PpAGO3^ΔRGG1-3^* mutants that carried a deletion of 1, 2, or 3 copies of the N-terminal RGG motif (QRGGYD) but failed to obtain complete knockout mutants, which suggests its vital role in *P. parasitica*. These mutants showed increased pathogenicity on both *Nicotiana benthamiana* and *Arabidopsis thaliana* plants. Transcriptome and sRNA sequencing of *PpAGO3^ΔRGG1^* and *PpAGO3^ΔRGG3^* showed that these mutants were differentially accumulated with 25–26 nt sRNAs associated with 70 predicted cytoplasmic effector genes compared to the wild-type, of which 13 exhibited inverse correlation between gene expression and 25–26 nt sRNA accumulation. Transient overexpression of the upregulated RXLR effector genes, *PPTG_01869* and *PPTG_15425* identified in the mutants *PpAGO3^ΔRGG1^* and *PpAGO3^ΔRGG3^*, strongly enhanced *N. benthamiana* susceptibility to *P. parasitica*. Our results suggest that PpAGO3 functions together with 25–26 nt sRNAs to confer dynamic expression regulation of effector genes in *P. parasitica*, thereby contributing to infection and pathogenicity of the pathogen.

## Introduction

Oomycetes represent a unique group of diploid microorganisms, which resemble but are evolutionarily distant from filamentous fungi (Judelson, 1997). Species in the genus of *Phytophthora* include many economically important plant pathogens, notably *Phytophthora infestans*, *P. sojae*, *P. parasitica*, and *P. ramorum* (Tyler, 2007; Fry, 2008; Grünwald et al., 2008).

During infection, *Phytophthora* secrets a large number of effectors into host plants, including RXLR (Arg–any amino acid-Leu-Arg) effectors and CRN (Crinkling and Necrosis) effectors, to manipulate host physiology and support colonization (Zheng et al., 2014; Fan et al., 2018; Wang and Wang, 2018; Chen et al., 2019). In response, the host plants produce R proteins to recognize the secreted effectors, which are known as avirulence proteins, triggering plant immunity. Nearly all host genotype-specific avirulence factors identified in *Phytophthora* are RXLR effectors (Armstrong et al., 2005; van Poppel et al., 2008; Bozkurt et al., 2011). At the same time, pathogens have evolved ways to evade the perception of effectors by the host R proteins through sequence variation and deletion of avirulence effectors. Apart from that, polymorphisms in the expression levels of effector genes are common in *Phytophthora* and contribute to pathogen plasticity in overcoming host genotype-specific resistance (Shan et al., 2004; Dong et al., 2009; Qutob et al., 2009; Wang et al., 2011a, 2020; Cui et al., 2012; Gijzen et al., 2014; Pais et al., 2018). Increasing evidence suggests that 25–26 nt small RNA (sRNA), the dominant size class of sRNAs in *Phytophthora*, are involved in the regulation of effector gene expression (Vetukuri et al., 2012; Qutob et al., 2013; Jia et al., 2017; Wang et al., 2019). For example, in *P. sojae*, 25 nt sRNAs are associated with *PsAvr3a* gene silencing and the silencing status could be “inherited” to the next generation (Qutob et al., 2013). In addition, up to 125 RXLR effector genes are associated with homologous sRNAs, which correlate with the silencing of the corresponding RXLR effector genes at the mycelium stage (Wang et al., 2019). Similarly, 25–26 nt sRNAs are implicated in the silencing of 40% RXLR and 50% CRN effector genes in *P. parasitica* during vegetative mycelial growth (Jia et al., 2017). However, very little is known about the mechanism and function of sRNA-associated silencing of RXLR effector genes.

The sRNA pathways are conserved in eukaryotes and play important roles in various cellular processes. Depending on the biogenesis pathway and functions, sRNAs are classified into microRNA (miRNA), small interfering RNA (siRNA), and PIWI-interacting RNAs (piRNAs; Girard et al., 2006; Carthew and Sontheimer, 2009; Ye et al., 2016). miRNAs are processed by Dicer or Dicer-like (DCL) proteins from self-folding RNA transcripts and have been widely found in plants and animals (Millar and Waterhouse, 2005), but very few miRNA-like genes exist in fungi and oomycetes (Lee et al., 2010; Fahlgren et al., 2013). piRNAs are only found in animals (Vagin et al., 2006; Brennecke et al., 2007; Czech et al., 2018), whereas siRNAs are present in most eukaryotes, and processed by Dicer or DCL from long double-stranded RNAs (dsRNAs) or hairpin structure (Katiyar-Agarwal et al., 2006; Huang et al., 2019b). To direct gene silencing, sRNAs are loaded to Argonaute (AGO) protein to form an RNA-induced silencing complex (RISC), which then uses the sRNA as guide to direct mRNA cleavage or DNA methylation/histone modification (Kobayashi and Tomari, 2016).

AGO proteins are present in both prokaryotes and eukaryotes (Bohmert et al., 1998; Makarova et al., 2009; Xue et al., 2012; Cervantes et al., 2013; Swarts et al., 2014; Nguyen et al., 2018; Yin et al., 2020), but the number and type of AGO proteins are diverse in different organisms (Tolia and Joshua-Tor, 2007; Hutvagner and Simard, 2008). There are primarily four AGO homologs, including AGO-like, PIWI, Worm-specific AGO (WAGO), and *Trypanosoma* AGO families (Durand-Dubief et al., 2003; Yigit et al., 2006; Hutvagner and Simard, 2008; Garcia–Silva et al., 2010; Bollmann et al., 2018). Members of the AGO-like family contain primarily the PAZ (PIWI–ARGONAUTE–ZWILLE) and PiWi domain, which contribute to the binding of sRNA 3′ end and the cleavage of complementary mRNA, respectively (Lingel et al., 2003; Song et al., 2004). The AGO-like family is primarily involved in miRNA and siRNA-directed silencing and plays a vital role in transcriptional and translational regulation, heterochromatin assembly, and alternative splicing (Baulcombe, 2004; Buker et al., 2007; Azzam et al., 2012; Wei et al., 2012; Marasovic et al., 2013; Huang and Li, 2014). The TbAGO1 in *Trypanosoma brucei* is required for RNAi and plays a role in mitosis and chromosome segregation (Durand-Dubief and Bastin, 2003; Garcia-Silva et al., 2010, 2014). Interestingly, besides the well-known PAZ and Piwi domains, TbAGO1 also contains an N-terminal RGG (arginine–glycine–glycine) repeat motif, which is required for RNA silencing (Shi et al., 2004). Loss of the N-terminal RGG domain can strongly block the association of TbAGO1 with polyribosomes and affect mRNA cleavage (Shi et al., 2004, 2009). The RGG domain of TgAGO in *Toxoplasma gondii* is also functional in the RNA silencing pathway (Musiyenko et al., 2012).

Several AGO proteins have been identified in *Phytophthora* and are implicated in sRNA-mediated regulation of effector genes. For example, in *P. infestans*, silencing of *PiAgo4* or *PiAgo5* caused reduced accumulation of 32 nt sRNAs homologous to *PiAvrblb1*, while silencing of *PiAgo1* resulted in increased accumulation of 32 nt sRNAs to *PiAvrblb1*. Interestingly, the *PiAvrblb1*-derived 32 nt sRNAs were not affected in *PiDCL1* silencing strain (Vetukuri et al., 2011, 2012). Furthermore, co-immunoprecipitation assays showed that CRN effector genes and pseudo-CRN-derived 18–30 nt sRNA were significantly enriched to PiAGO1 and PiAGO5 proteins (Åsman et al., 2016). However, little is known on the specific roles of AGOs in *Phytophthora* biology and pathology.

In this study, we investigated the function of *PpAGO3* in the model oomycete organism *P. parasitica* (Meng et al., 2014), where the efficient, complete silencing of many effector genes is associated with accumulation of their homologous 25–26 nt sRNAs during the mycelium stage (Jia et al., 2017). We created mutations in the N-terminal RGG domain repeat region of PpAGO3 by using the CRISPR-Cas9 technology. PpAGO3 mutants (*PpAGO3^ΔRGG1-3^*), with the deletion of 1–3 copies of RGG domain (QRGGYD), showed enhanced pathogenicity and examined for sRNA accumulation and gene expression. In addition, two PpAGO3/sRNA-regulated RXLR effector genes were further analyzed for their virulence function. Our results provide compelling evidence that *PpAGO3* and 25–26 nt sRNAs function together to regulate effector gene expression.

## Materials and Methods

### Plant Growing and *Phytophthora parasitica* Cultivation

*Arabidopsis thaliana* and *Nicotiana benthamiana* seeds were sown in a matrix containing soil and vermiculite, and cultured in a phytotron (23°C) with a photoperiod of 14 h light per day for about 4–5 weeks. *Phytophthora parasitica* strain PpBS042 was isolated from diseased tobacco plant collected from Chongqing, China (Zhang et al., 2020). It was routinely cultured on 5% CA (carrot juice agar) medium with 0.01% CaCO_3_ and 0.002% β-sitosterol, in darkness for 3–4 days (23°C). To induce the sporangia production, the culture medium with the fresh mycelia were transformed into 5% CA broth with Petri solution for another 5 days as described before (Huang et al., 2019a). For the zoospore release, it was done as described previously (Wang et al., 2011b).

### Bioinformatics Analysis

The five AGO protein sequences of *P. infestans* were BLASTP aligned with the *P. parasitica* INRA-310 (taxid:761204) database on NCBI, and the five predicted AGO proteins sequences of *P. parasitica* were BLASTP aligned to *P. infestans* T30-4 (taxid:403677) database to ensure the consistency of the sequence alignment. The sequence ID of five AGO proteins in *P. infestans* were PiAGO1 (XP_002906080.1), PiAGO2 (XP_002906081.1), PiAGO3 (XP_002908068.1), PiAGO4 (XP_002908108.1), PiAGO5 (XP_002908109.1). The domain architecture was predicted by using Pfam database and displayed by software IBS (Liu et al., 2015). For gene expression pattern analysis, it was conducted by using the RNA-seq data obtained by Jia (2017), and the RNA-seq data analysis would be described later.

### Genome Editing in *Phytophthora parasitica* by CRISPR-Cas9 System

The *PpAGO3* mutants were obtained through three main steps: CRISPR-Cas9 vector construction for gene *PpAGO3*; the plasmid transformation in *P. parasitica*; transformants selection and identification. Firstly, the CRISPR-Cas9 plasmid was constructed and modified using “all-in-one” vector pYF515 developed for *P. sojae* (Fang et al., 2017). The sgRNA for *PpAGO3* was designed by using EuPaGDT^1^; then, off-target was analyzed by using FungiDB^2^; finally, RNA secondary structure was predicted online.^3^ For *PpAGO3*, one sgRNA (total score > 0.5) without off-target site and weak RNA secondary structure was chosen for primer design. Through annealing and extension, the synthesized primers were made to dsDNA fragment and were ligated to NheI/BsaI (NEB) digested vector PYF515 by using T4 DNA ligase (Promega). The recombination vector was then transformed into *Escherichia coli* DH5α cells. The sequence-verified plasmid was extracted and concentrated for transformation.

Secondly, CRISPR-Cas9 plasmid for *PpAGO3* was transformed into *P. parasitica* strain *PpBS042*. The transformation was conducted by using PEG-CaCl_2_ mediated method as described (Bottin et al., 1999; Meng et al., 2015). The transformation protoplasts were recovered overnight and then were cultured on the 5% CA medium with 13.6 μg/ml G418, 200 μg/ml ampicillin, 20 μg/ml nystatin, and 20 μg/ml rifampicin. Finally, it was the selection and identification for *PpAGO3* mutants. Through 3–7 days selective cultivation (23°C), regenerated mycelium colonies were isolated and transformed to a fresh plate with the same selective medium. Three days later, the G418-resistant transformants were cultivated in 5% CA broth for mycelium collection. Genomic DNA was extracted following the protocol as described previously (Zhang et al., 2020) from each candidate transformants and was examined for target sites by sequence amplification and sequencing.

### Pathogenicity Assay

*Phytophthora parasitica* was cultivated on 5% CA plates as described (Huang et al., 2019a; Zhang et al., 2020). Detached leaves of the 5-week-old *N. benthamiana* and 4-week-old *A. thaliana* Col-0 leaves were used for pathogenicity assays as described previously (Huang et al., 2019a; Zhang et al., 2020). On *N. benthamiana* leaves, the developed lesions were measured 60 h post-inoculation with *P. parasitica* mycelial discs, and the expansion of *P. parasitica* hyphae was visualized by trypan blue staining. For each assay, more than eight leaves were used. On *A. thaliana*, the disease severity index (DSI) was recorded 48 h after inoculation with *P. parasitica* mycelial discs, as described previously (Huang et al., 2019a). For each assay, more than 15 leaves were used. Statistical analysis was performed based on Student’s *t*-test between samples and based on a one-way ANOVA.

### RNA Extraction, Library Construction, and Sequencing

For library construction, *P. parasitica* strain *PpBS042* was firstly cultured on 5% CA solid medium with 0.01% CaCO_3_ and 0.002% β-sitosterol, in darkness for 3–4 days (23°C). Then, the culture medium with the fresh mycelia were transformed into 5% CA broth with Petri solution for another 3 days. The total RNA was extracted from fresh mycelia of both the wild-type and *PpAGO3^ΔRGG^* mutants by using the RNA extract kit (Aidlab, RN40). The RNA concentration and quality were examined by using NanoDrop 2000 and gel electrophoresis. Construction and sequencing of small RNA and RNA libraries were performed by Biomarker Technologies (Beijing, China) with Illumina novaseq 6000 platform. For construction of sRNA library, only 18–45 nt size small RNAs were used for sequencing. The wild-type and the two *PpAGO3^ΔRGG^* mutants contain three biological replicates, respectively.

### High-Throughput Sequencing Data Analysis

According to sRNA raw data, adaptor sequence (AGATCGGAAGAGCACACGTCTG) was first filtered. Then, clean reads were mapped to the *P. parasitica* genome (INRA-310 version 3.0, Assembly Dev initiative, Broad Institute) with Bowtie with no mismatches (-v 0 -a; Langmead et al., 2009; Jia et al., 2017). Reads mapped to rRNA, tRNA, mitochondrial DNA were also filtered by using bowtie (-v 0). The sRNAs that mapped to the gene locus (gene body plus 500 bp upstream and downstream region) were counted and regarded as the sRNA accumulation. Depending on the strand (sense/antisense) the sRNAs derived and the sRNA size, the detailed analysis was further divided. The sRNA counts were calculated by using self-write perl scripts. For sRNAs differentially expressed between the wild-type and *PpAGO3^ΔRGG^* mutants, the mapping counts were analyzed by using R package, DESeq2 (Love et al., 2014). For each gene, the corresponding sRNA expression level was calculated by using RPKM, reads Per Kilobase per Million.

According to the RNA-seq raw data, the adapter sequence and low-quality reads were filtered by using software Trimmomatic (-phred33 LEADING:3 TRAILING:3 SLIDINGWINDOW:4:15 MINLEN:36; Bolger et al., 2014). The clean reads were mapped to *P. parasitica* genome (INRA-310-version 3.0) by using software Hisat2 (-min-intronlen 20 -max-intronlen 3,000; Kim et al., 2015). Software featureCounts was used for mapping counts calculation (Liao et al., 2014). The differential expression analysis was conducted by using DESeq2 (Love et al., 2014). Gene annotation of differentially expressed genes (DEGs) was done by Blast2go (Conesa et al., 2005), and GO enrichment analysis was realized by using software Tbtools (Chen et al., 2020). FeatureCounts output data were used to calculate gene expression level, FPKM. The heatmap for gene/sRNA expression level was performed by using Tbtools (Chen et al., 2020). The expression pattern of *PpAGO* family genes was obtained by analyzing the RNA-seq data obtained previously (Jia, 2017). The RNA sequencing was conducted by collecting the 6-week-old *N. benthamiana* leaves infected with *P. parasitica* strain Pp016 at 3, 6, 12, 24, and 48 h post-inoculation (Jia, 2017). The FPKM values were calculated and extracted by using Cuffquant and Cuffnorm (Trapnell et al., 2012).

### Vector Construction and *Agrobacterium tumefaciens*-Mediated Transient Expression Assay

RXLR effector genes PPTG_01869 and PPTG_15425 were fused with *GFP*. The effector gene fragment and *GFP* were both amplified by using *P. parasitica* cDNA and DNA polymerase FastPfu (TransGen Biotech). The generated fragments were ligated to the restriction sites (KpnI and XbaI) in the vector pKannibal (Wesley et al., 2001) by using T4 DNA ligase (Promega). The fusion construct was further transferred to the *Not*I sites in vector pART27 (Gleave, 1992). The resulted constructs *PPTG_01869*-*GFP*, *PPTG_15425*-*GFP*, and *GFP* were transferred into *Agrobacterium tumefaciens* GV3101 cells, respectively, and cultured in Luria Bertani (LB) liquid broth, harvested and suspended in infiltration buffer as described (Huang et al., 2019a). The agroinfiltration was performed at concentration (OD_600_ 0.04) on *N. benthamiana* leaves by using needleless syringes (Meng et al., 2015). The infiltrated *N. benthamiana* leaves were detached and gene expression state was examined by using the fluorescence microscope (Olympus-BX-51TRF) 24–36 h post-infiltration, and pathogenicity assays were performed using procedures as described (Huang et al., 2019a). Eight leaves were used for each experiment and the experiments were repeated three times.

## Results

### Mutations in PpAGO3 Led to Enhanced Pathogenicity of *Phytophthora parasitica*

*Phytophthora* genomes encode several AGO homologs that phylogenetically clustered with the AGO-like family (Åsman et al., 2016; Bollmann et al., 2016, 2018). In *P. parasitica*, five AGO proteins, designated as PpAGO1-5 (Supplementary Figure 1), were shown to have high sequence homologies to PiAGOs of *P. infestans*, a sister species of *P. parasitica*. Searching the Pfam database (Finn et al., 2010) revealed that they all contain the typical domains of AGO, including the N-terminal PAZ, Mid (middle), and Piwi domains with one or two linkers (L1 and L2; Supplementary Figure 1). In addition, they all contain the arginine–glycine–glycine (RGG) repeat motif at the N terminus. However, the number of RGG motif is highly variable among five PpAGO proteins, with one for PpAGO1, three for PpAGO2, 24 for PpAGO3, two for PpAGO4, and 13 for PpAGO5 (Supplementary Figure 1).

We examined the expression pattern of five *PpAGO* genes using RNA-seq data of *P. parasitica* Pp016 before and after infection of *N. benthamiana* (Jia, 2017). The results showed that *PpAGO3* is highly expressed in mycelia (Supplementary Figure 1) during which the class of the 25–26 nt sRNA is abundant (Jia et al., 2017).

**Figure 1:**
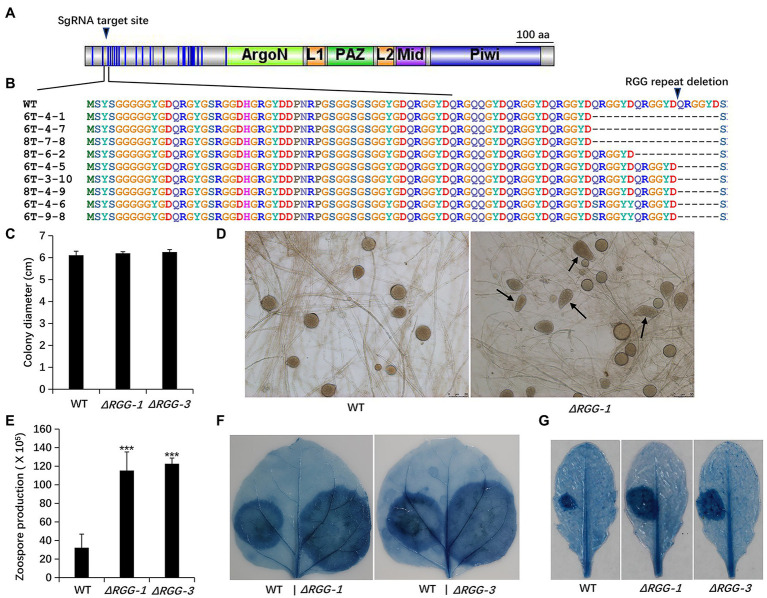
Generation and phenotypic analysis of *Phytophthora parasitica* mutants *PpAGO3^ΔRGG1^* and *PpAGO3^ΔRGG3^*. **(A)** The designed sgRNA targeting sites on PpAGO3 protein. **(B)** Mutants *PpAGO3^ΔRGG1^* and *PpAGO3^ΔRGG3^* were generated by CRISPR-Cas9 technology. Three types of mutation were obtained: an 18 aa (amino acids) deletion (three QRGGYD repeats) in mutants 6T-4-1, 6T-4-7, and 8T-7-8; a 12 aa deletion (two repeats) for mutant 8T-6-2; and a six aa deletion (one repeat) for mutants 6T-4-5, 6T-3-10, 8T-4-9, 6T-4-6, and 6T-9-8. Besides that, a single amino acid substitution within RGG motif was notable for mutants 6T-4-6 and 6T-9-8. Mutants *PpAGO3^ΔRGG1^* (*ΔRGG-1*) and *PpAGO3^ΔRGG3^* (*ΔRGG-3*) showed similar growth rate to the wild-type, as shown by *t*-test **(C)**, produced abnormal sporangium **(D)** and more zoospores than the wild-type (Student test: ^***^p < 0.001) **(E)**, and caused larger lesions than the wild-type on *Nicotiana benthamiana* **(F)** and on *Arabidopsis thaliana* **(G)**. The detached 5-week-old *N. benthamiana* and 4-week-old *A. thaliana* leaves were inoculated with mycelial discs and the water-soaked lesions were examined by trypan blue staining at 48 hpi. Similar results were obtained from three independent experiments.

To investigate the potential biological function of *PpAGO*, we employed CRISPR-Cas9 gene editing system (Fang et al., 2017; Zhang et al., 2020) to mutate *PpAGO3*. The N-terminal sequence of PpAGO3 contains 24 copies of the RGG motif. Through two independent transformation experiments, we obtained nine *PpAGO3* mutants all with three types of mutations that carry a deletion of 1–3 copies of the RGG domain in the N terminus without frame shifts or complete knockout (Figures 1A,B), suggesting PpAGO3 is vital to *P. parasitica*.

Phenotypic analysis showed that the *PpAGO3^ΔRGG^* mutants displayed stronger growth vigor than the wild-type strain, showing more dense and thicker mycelia on 5% CA agar plates, though the colony diameters remained unchanged (Figure 1C). In addition, *PpAGO3^ΔRGG^* mutants always produce some abnormal sporangia (Figure 1D) and released many more zoospores than wild-type strain (Figure 1E). More interestingly, *PpAGO3^ΔRGG^* mutants were more invasive than the wild-type *P. parasitica* on *N. benthamiana* leaves (Figure 1F). Similarly, *A. thaliana* leaves infected with *PpAGO3^ΔRGG^* mutants also showed stronger disease phenotypes (Figure 1G). These results suggest that *PpAGO3* plays vital role in the pathogenicity and development of *P. parasitica*.

### The Expression of 25–26 nt sRNAs Derived From Effector Genes Is Significantly Changed in the Mutants *PpAGO3^ΔRGG1^* and *PpAGO3^ΔRGG3^*

Our previous study showed that the 25–26 nt sRNAs are enriched at the vegetative mycelium stage and is associated with high level of silencing of numerous effector genes in *P. parasitica*, including 40% RXLR (226) and 50% CRN (147) effector genes (Jia et al., 2017). The high level of *PpAGO3* expression in vegetative hyphae, together with the enhanced virulence of the *PpAGO3^ΔRGG^* mutants, led us to assume that PpAGO3 may interact with 25–26 nt sRNAs to regulate expression of effector genes. Small RNA sequencing was therefore conducted by using RNA isolated from fresh hyphae tissues of the wild-type and mutants *PpAGO3^ΔRGG1^* and *PpAGO^ΔRGG3^*.

**Figure 2:**
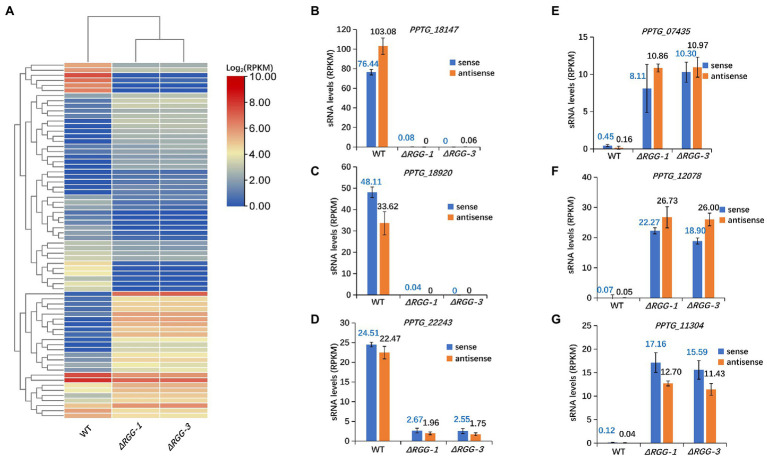
Significant changes in accumulation of 25–26 nt sRNAs homologous to70 cytoplasmic effector genes in mutants *PpAGO3^ΔRGG1^* and *PpAGO3^ΔRGG3^* compared to the wild-type *P. parasitica*. **(A)** Heatmap for 25–26 nt sRNA accumulation in wild-type, and mutants *PpAGO3^ΔRGG1^* and *PpAGO3^ΔRGG3^*. The accumulation of 25–26 nt sRNAs was increased for those associated with 24 RXLR and 17 CRN effector genes, and was reduced for those associated with 12 RXLR and 17 CRN effector genes in mutants *PpAGO3^ΔRGG1^* and *PpAGO3^ΔRGG3^*. The color represents the transformation value of log_2_ (RPKM). **(B-G)** The expression levels of sense and antisense 25–26 nt sRNAs associated with six representative effector genes in wild-type, and mutants *PpAGO3^ΔRGG1^* and *PpAGO3^ΔRGG3^*. Both sense and antisense 25–26 nt sRNAs associated *PPTG_18147* **(B)**, *PPTG_18920* **(C),** and *PPTG_22243* **(D)** were highly accumulated in the wild-type but reduced dramatically in mutants *PpAGO3^ΔRGG1^* and *PpAGO3^ΔRGG3^*. On the contrary, the sense and antisense 25–26 nt sRNAs associated with *PPTG_07435* **(E)**, *PPTG_12078* **(F)**, and *PPTG_11304* **(G)** were significantly increased in *PpAGO3^ΔRGG1^* and *PpAGO3^ΔRGG3^* mutants compared to the wild-type.

Clean reads of 18–45 nt sRNAs that mapped to *P. parasitica* genome (*P. parasitica INRA-310* version 3.0, Assembly Dev initiative, Broad Institute)^4^ excluding the rDNA, mtDNA, and tRNA sequences, were obtained for further analysis. The overall sRNA counts and 25–26 nt sRNA percent did not seem to be affected in mutants *PpAGO3^ΔRGG1^* and *PpAGO3^ΔRGG3^* (Supplementary Table 1). Importantly, this analysis detected differential accumulation of the 25–26 nt sRNAs specific to some RXLR and CRN effector genes (Supplementary Table 2) compared to the wild-type strain. Of the 226 RXLR effector genes, 36 (15.9%) contained differentially accumulated 25–26 nt sRNAs in the gene body and 500 nt upstream and downstream flanking sequences in mutants *PpAGO3^ΔRGG1^* and *PpAGO3^ΔRGG3^*, of which 24 genes showed increased and 12 decreased sRNA accumulation (Figure 2A). For the 147 predicted CRN effector genes, 34 (23.13%) displayed differential 25–26 nt sRNA accumulation in the mutants compared to the wild-type strain (Supplementary Table 2), with 17 showing increased and 17 reduced sRNA accumulation (Figure 2A).

Our previous studies showed that most of sRNAs in *P. parasitica* are derived from both sense and antisense strands of the genes typical of double-stranded RNA (dsRNA) processing (Jia et al., 2017). For 49 of the 70 effector genes (36 RXLR and 34 CRN effector genes), 25–26 nt sRNAs of both sense and antisense polarities were differentially expressed in the *PpAGO3^ΔRGG^* mutants compared to the wild-type strain. For example, the effector genes *PPTG_18147*, *PPTG_18920*, and *PPTG_22243*, showed a large number of 25–26 nt sense and antisense sRNA in the wild-type strain, but these 25–26 nt sRNAs could not be detected in mutants *PpAGO3^ΔRGG1^* and *PpAGO3^ΔRGG3^* (Figures 2B–D). On the contrary, both the sense and antisense 25–26 nt sRNAs matching the effector genes *PPTG_07435*, *PPTG_12078*, and *PPTG_11304*, showed increased accumulation in the mutants (Figures 2E–G). These results indicated that PpAGO3 is involved in the accumulation of 25–26 nt sRNAs in *P. parasitica* that have both sense and antisense polarities typical of dsRNA-derived sRNAs.

### Expression Changes of 25–26 nt sRNAs Associated With Non-effector Genes in the Mutants *PpAGO3^ΔRGG1^* and *PpAGO3^ΔRGG3^*

**Figure 3:**
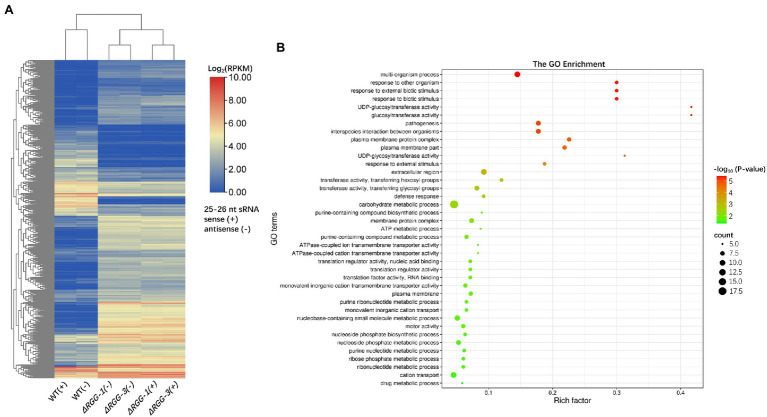
The sRNA distribution of non-effector genes in mutants *PpAGO3^ΔRGG1^* and *PpAGO3^ΔRGG3^*. **(A)** Both sense (+) and antisense (−) strand-derived 25–26 nt sRNAs associated with 1808 non-effector genes were differentially accumulated in mutants compared to the wild-type. RPKM, Reads Per Kilobase per Million mapped reads; the heatmap color represents the transformation value of log_2_ (RPKM). **(B)** The GO enrichment analysis for genes with which 25–26 nt sRNAs were downregulated in mutants *PpAGO3^ΔRGG1^* and *PpAGO3^ΔRGG3^*. In the bubble plot, *x*-axis represents the rich factor; the *y*-axis represents the enriched terms; the bubble size represents the enriched gene count. The larger the bubble is, the more genes enriched; the bubble color represents the enriched significance which depends on the data of –log_10_ (value of *p*). GO enrichment was conducted by using software Tbtools and visualized online: http://www.bioinformatics.com.cn/.

In addition to effector genes, differential accumulation of 25–26 nt sRNA was also observed for 2,508 non-effector genes. Of these genes, 1,607 showed increased sRNA accumulation and 901 showed reduced accumulation (Supplementary Table 2). The differentially accumulated sRNAs from 1808 genes contained both sense and antisense populations (Figure 3A), which provided more evidence that these differentially expressed sRNAs are generated from dsRNA and associated with PpAGO3 protein.

GO enrichment analysis of these 2,508 non-effector genes showed that the downregulated sRNAs were mainly associated with genes in the Biological Process level category, including multi-organism process, response to external biotic stimulus, response to biotic stimulus and to other organism, pathogenesis, and interspecies interaction between organism terms (Figure 3B). The association of differentially accumulated 25–26 nt sRNAs with biotic response and pathogenesis genes, in addition to effector genes, further suggest the importance of these sRNAs in the regulation of *Phytophthora* pathogenesis.

### Upregulated Effector Genes in the Mutants *PpAGO3^ΔRGG1^* and *PpAGO3^ΔRGG3^*

The expression variation of effector gene-associated 25–26 nt sRNAs in *PpAGO3^ΔRGG^* mutants and increased virulence of the mutants prompted us to examine if expression of effector genes were influenced. RNA sequencing (RNA-seq) was performed on wild-type and mutants *PpAGO3^ΔRGG1^* and *PpAGO3^ΔRGG3^* using RNA samples extracted from fresh mycelia. Analysis of the RNA-seq data revealed that 24 RXLR effector genes, 10.6% of the 226 predicted RXLR effector genes in *P. parasitica*, were differentially expressed (|log_2_ Fold change| > 1 and Padj <0.05; Supplementary Table 3). Of these 24 genes, 12 showed increased mRNA level while the other 12 reduced mRNA level (Supplementary Table 3). For the CRN effector genes, 18.4% (27/147) genes exhibited differential expression with 17 being upregulated and 10 downregulated (Supplementary Table 3). In total, 51 effector genes were differentially expressed in the *PpAGO3^ΔRGG^*mutants compared to the wild-type strain.

**Figure 4:**
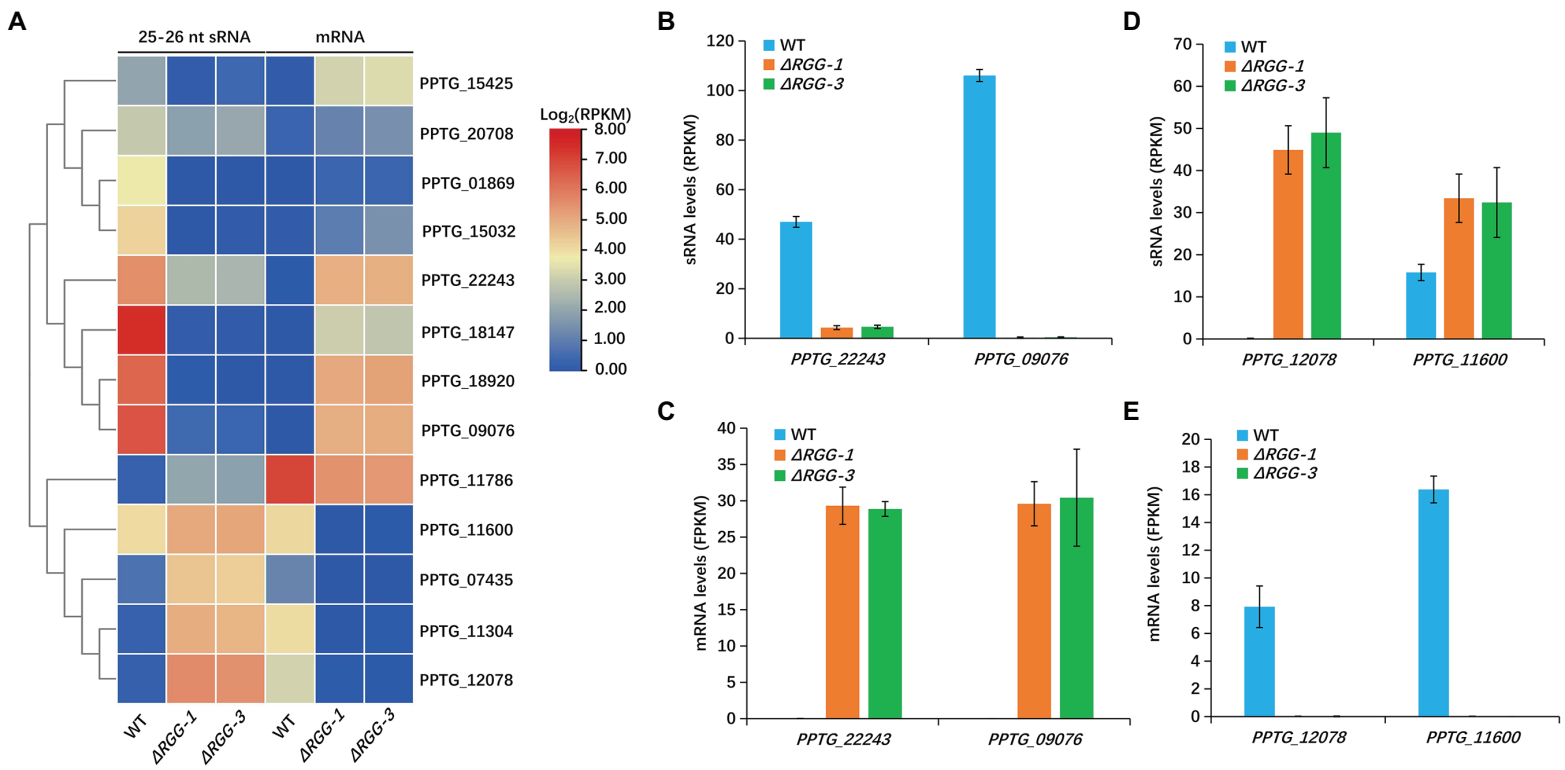
The changed 25–26 nt sRNA accumulation in mutants *PpAGO3^ΔRGG1^* and *PpAGO3^ΔRGG3^* was negatively correlated with expression of the corresponding effector genes. **(A)** The heatmap for 13 effector genes with which 25–26 nt sRNA and mRNA levels were negatively correlated in mutants *PpAGO3^ΔRGG1^* and *PpAGO3^ΔRGG3^* compared to the wild-type. The color represents the transformation value of log_2_(RPKM) for sRNA and log_2_(FPKM) for mRNA. **(B–E)** The 25–26 nt sRNA and mRNA levels of four representative effector genes, *PPTG_22243*, *PPTG_09076*, *PPTG_12078*, and *PPTG_11600*. The 25–26 nt sRNA levels were significantly reduced for *PPTG_22243* and *PPTG_09076* **(B)** and the mRNA levels were increased obviously **(C)** in mutants *PpAGO3^ΔRGG1^* and *PpAGO3^ΔRGG3^*. On the contrary, the 25–26 nt sRNA levels were increased **(D)** but the mRNA levels were reduced **(E)** for *PPTG_12078* and *PPTG_11600*.

We then investigated whether the effector genes with differential mRNA level were associated with differentially accumulated 25–26 nt sRNAs. Overlapping the 51 differentially expressed effector genes (Supplementary Table 3) with the 70 effector genes that had differentially accumulated 25–26 nt sRNAs (Figure 2A) identified 14 effector genes that showed significant changes in both 25–26 nt sRNA accumulation and mRNA level. Interestingly, except for *PPTG_01844*, the remaining 13 effector genes, including 4 RXLR and 9 CRN effector genes, all showed a negative correlation between sRNA accumulation and mRNA expression level (Figure 4A). In particular, 8 genes showed reduced accumulation of 25–26 nt sRNA that correlated with increased gene expression level in *PpAGO3^ΔRGG^* mutants compared to the wild-type strain, whereas five genes showed increased sRNA accumulation with reduced gene expression (Figure 4A). For instance, *PPTG_22243* and *PPTG_09076* both showed a dramatic reduction in 25–26 nt sRNA abundance in *PpAGO3^ΔRGG^* mutants (Figure 4B), which correlated with strong upregulation of gene expression at the transcript level (Figure 4C). On the contrary, *PPTG_12078* and *PPTG_11600* showed an increase in 25–26 nt sRNA level but with reduction on mRNA level (Figures 4D,E). Taken together, these results suggest that PpAGO3 interacts with 25–26 nt sRNA to regulate the expression of the sRNA-associated effector genes.

### The PpAGO3-Regulated RXLR Effector Genes *PPTG_01869* and *PPTG_15425* Both Contribute to *Phytophthora parasitica* Virulence

**Figure 5:**
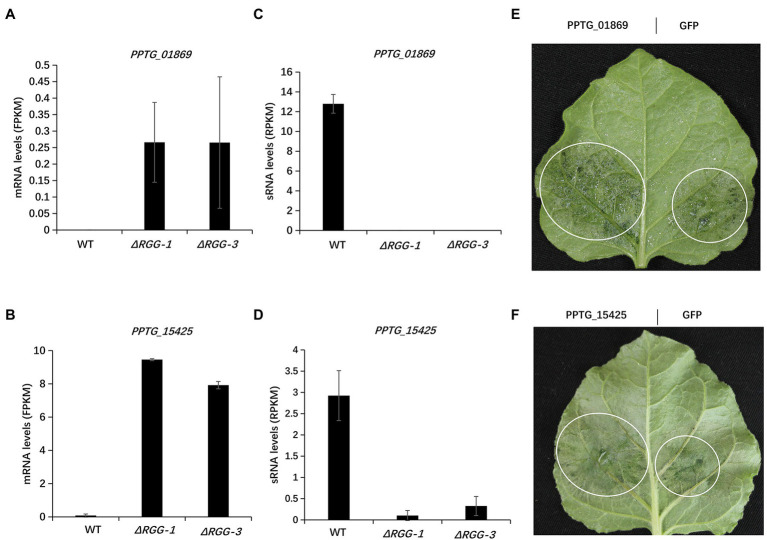
The enhanced pathogenicity of mutants *PpAGO3^ΔRGG1^* and *PpAGO3^ΔRGG3^* was likely resulted from activation of effector genes. **(A,B)** The mRNA levels of two upregulated RXLR effector genes, *PPTG_01869* and *PPTG_15425*, in *PpAGO3^ΔRGG1^* and *PpAGO3^ΔRGG3^* mutants. **(C,D)** The changes of *PPTG_01869* and *PPTG_15425* mRNA levels were negatively with the corresponding 25–26 nt sRNA levels variation in *PpAGO3^ΔRGG1^* and *PpAGO3^ΔRGG3^* mutants. **(E,F)** *Phytophthora parasitica* caused much larger lesion on *PPTG_01869* and *PPTG_15425* transient expressed *N. benthamiana* leaves than control *GFP*, respectively. The white circle marked the water-soaked lesion region. Similar results were obtained from three independent experiments.

We next examined if these upregulated effector genes contribute to the enhanced pathogenicity of the mutants *PpAGO3^ΔRGG1^* and *PpAGO3^ΔRGG3^*, by analyzing the virulence function of the effector genes using *A. tumefaciens* infiltration-delivered transient expression. We selected two RXLR effector genes, *PPTG_01869* and *PPTG_15425*, which showed strong upregulation of mRNA levels in *PpAGO3^ΔRGG^* mutants with dramatic reduction of 25–26 nt sRNA accumulation (Figures 5A–D). *Nicotiana benthamiana* leaves infiltrated with the *PPTG_01869-*overexpression construct and inoculated with *P. parasitica* mycelial disks developed significantly larger lesions at 2 days post-inoculation than leaves infiltrated with the control GFP construct (Figure 5E). Similarly, *A. tumefaciens*-mediated overexpression of *PPTG_15425* strongly enhanced *P. parasitica* infection (Figure 5F). These results indicated that the *PpAGO3*-regulated *PPTG_01869* and *PPTG_15425* effector genes play a positive role in *P. parasitica* virulence.

## Discussion

A number of reports have suggested that sRNA pathways participate in the silencing of effector genes in *Phytophthora* species (Vetukuri et al., 2012; Qutob et al., 2013; Jia et al., 2017; Wang et al., 2019). AGO proteins, as the key component of RNAi pathway, were reported to be associated with effector gene-derived sRNA (Vetukuri et al., 2011, 2012; Åsman et al., 2016). These findings suggested the potential function of AGO proteins and sRNAs in the effector genes regulation in *Phytophthora* but the real functional role of the RNA silencing pathways in *Phytophthora* pathogenicity has yet to be confirmed.

In this study, we used the model organism *P. parasitica* to examine the function of AGO in effector gene regulation and pathogenicity. Through sequence analysis, five AGO proteins were identified which all consisted of the conserved domains, PAZ, Mid and Piwi. Functional studies focused on PpAGO3, a unique member with 24 RGG repeats at the beginning of the N terminus and highly expressed in vegetative hyphae.

Evidence for the involvement of PpAGO3 in the pathogenicity of *P. parasitica* was first suggested by the increased growth vigor of the mutants *PpAGO3^ΔRGG1-3^* deleted with 1–3 copies of the RGG domain, which were generated using the CRISPR-Cas9 system. These mutants displayed increased growth on medium and produced abnormal sporangia and greatly increased number of zoospores. More significantly, the *PpAGO3^ΔRGG^* mutants were much more invasive in the tested host plants *N. benthamiana* and *A. thaliana* than the wild-type strain.

AGO proteins regulate gene expression through interactions with sRNAs. Consistent with this, our sRNA sequencing analysis of the mutants *PpAGO3^ΔRGG1^* and *PpAGO3^ΔRGG3^* both detected altered accumulation of 25–26 nt sRNAs homologous to 70 effector genes, and 13 of these effector genes showed an inverse correlation between their expression levels and the abundance of the associated 25–26 nt sRNAs. The role of PpAGO3 in regulating effector gene expression is further supported by the opposite developmental stage expression profiles of the PpAGO3 and some of the effector genes. Three such effector genes showed low expression levels at the mycelium stage where the level of *PpAGO3* expression was the highest. In contrast, these genes were induced during the infection where the expression of *PpAGO3* is reduced.

The functional significance of PpAGO3 regulation of effector genes was shown by the transient overexpression assay of two PpAGO3-regulated effector genes, *PPTG_01869* and *PPTG_15425*. These two genes were strongly upregulated in the *PpAGO3^ΔRGG^* mutants, which correlated inversely with reduced abundance of 25–26 nt sRNAs. Overexpression of both of these two effector genes, using agrobacterium infiltration in *N. benthmiana* leaves, enhanced the infection of *P. parasitica*. This result suggests that the enhanced virulence of the *PpAGO3^ΔRGG^* mutants is related to the activated expression of the sRNA-regulated effector genes. Taken together, our results provided compelling evidence that PpAGO3, in conjunction with 25–26 nt sRNAs, plays a major role in the regulation of effector gene expression in *P. parasitica*, and this regulation contributes to the virulence of the pathogen in host plants. However, how PpAGO3 interact with 25–26 nt sRNAs and how the effector genes are regulated, remain to be further studied.

One question is why these effector genes need to be regulated by the sRNA silencing pathways. Here we propose three possible scenarios. First, it was considered that some effectors could trigger plant immunity as avirulence factors, and suppression of these effectors would help to evade the perception by host plants. Consistently, some avirulence effector genes are suppressed in *P. infestans* and *P. sojae* (Shan et al., 2004; Wang et al., 2019), and the silencing of *PiAGO1*, *PiAGO4*, or *PiAGO5* in *P. infestans* is associated with the accumulation of *PiAvrblb1* homologous 32 nt sRNA (Vetukuri et al., 2011, 2012). Second, some effectors might be super-virulent to host plants, and silencing of these effector genes is required to establish a balance between successful infection and continuous evolution in the host. The last possible scenario is that, in order to conserve energy, some effectors are suppressed at the mycelium stage but only derepressed when needed during the infection process, like the effector genes *PPTG_01869* and *PPTG_15425* shown in this study. Further studies are needed to investigate these hypotheses.

Besides the predicted effector genes, PpAGO3 appears to regulate many other genes in *P. parasitica*. The RNA-seq analysis identified a total of 3,047 genes with differential expression in *PpAGO3^ΔRGG^* mutants (Supplementary Table 3), and sRNA-seq data detected 2,578 genes with differential accumulation of 25–26 nt sRNA (Supplementary Table 2). In total, across the *P. parasitica* genome, 509 non-cytoplasmic effector genes were identified to show negative correlation between 25 and 26 nt sRNA abundance and mRNA level (Supplementary Figure 2A). These genes include three INF-like genes (Supplementary Figures 2B,C), one of which could induce cell death shown by our unpublished work. The function of these non-cytoplasmic effector genes in *Phytophthora*–host plant interactions is worth further investigation.

Some of the genes downregulated in *PpAGO3^ΔRGG^* mutants were associated with increased abundance of 25–26 nt sRNA accumulation, suggesting a role of these sRNAs in the high-level silencing of these genes. It is possible that other AGO members also interact with the 25–26 nt sRNAs, and in the *PpAGO3^ΔRGG^* mutants, these AGOs function to repress the expression of these genes. Future studies should examine the interactions of 25–26 nt sRNAs with the different AGO members in *P. parasitica*.

For AGO proteins, a diverse number of RGG motifs was found localized in the N-terminal region of each PpAGO proteins (Supplementary Figure 1). The N-terminal RGG domain was reported to play an important role in AGO-mediated RNA silencing in *T. brucei* and *T. gondii* (Shi et al., 2004, 2009; Musiyenko et al., 2012), and is also important for the function of other RGG-containing proteins in pre-mRNA splicing, transcription and mRNA translation processes (Rajyaguru and Parker, 2012; Thandapani et al., 2013; Chong et al., 2018). In this study, our results suggested that the RGG domain is also functionally important in *P. parasitica*.

In conclusion, the RGG domain-changed *PpAGO3^ΔRGG^* mutants have allowed us to generate compelling evidence that PpAGO3 plays an important role in regulating effector gene expression in *P. parasitica* through interaction with the 25–26 nt class of sRNAs. Future studies should investigate the interactions between PpAGO3 and the 25–26 nt sRNAs and how the 25–26 nt sRNAs are generated and how they direct silencing of the effector genes. Moreover, a detailed functional analysis of the RGG and other domains as well as the full-length protein of PpAGO3 were also necessary, especially for the role in *Phytophthora*–host plant interactions.

## Data Availability Statement

The datasets presented in this study can be found in online repositories. The names of the repository/repositories and accession number(s) can be found in the article/Supplementary Material.

## Author Contributions

WS, YM, and JX designed the experiment. JX, YL, JJ, WX, CZ, GH, XG, and YM performed the experiment. JX, WS, JJ, and CZ analyzed the data. JX and WS wrote the paper with suggestions from all authors. All authors contributed to the article and approved the submitted version.

## Funding

This study received funding from National Natural Science Foundation of China (31561143007), China Agriculture Research System (CARS-09), and the State Administration of Foreign Experts Affairs (#B18042). The funder was not involved in the study design, collection, analysis, interpretation of data, the writing of this article, or the decision to submit it for publication.

## Conflict of Interest

The authors declare that the research was conducted in the absence of any commercial or financial relationships that could be construed as a potential conflict of interest.

## Publisher’s Note

All claims expressed in this article are solely those of the authors and do not necessarily represent those of their affiliated organizations, or those of the publisher, the editors and the reviewers. Any product that may be evaluated in this article, or claim that may be made by its manufacturer, is not guaranteed or endorsed by the publisher.

## Footnotes

^1^ http://grna.ctegd.uga.edu/

^2^ https://fungidb.org/fungidb/

^3^ http://rna.urmc.rochester.edu/RNAstructureWeb/Servers/Predict1/Predict1.html

^4^ https://www.broadinstitute.org/

## References

[ref1] Armstrong M. R. Whisson S. C. Pritchard L. Bos J. I. Venter E. Avrova A. O.. (2005). An ancestral oomycete locus contains late blight avirulence gene *Avr3a*, encoding a protein that is recognized in the host cytoplasm. Proc. Natl. Acad. Sci. U.S.A. 102, 7766–7771. doi: 10.1073/pnas.0500113102, PMID: 15894622 PMC1140420

[ref2] Åsman A. K. Fogelqvist J. Vetukuri R. R. Dixelius C. (2016). *Phytophthora infestans* Argonaute 1 binds microRNA and small RNAs from effector genes and transposable elements. New Phytol. 211, 993–1007. doi: 10.1111/nph.13946 27010746

[ref3] Azzam G. Smibert P. Lai E. C. Liu J. L. (2012). Drosophila Argonaute 1 and its miRNA biogenesis partners are required for oocyte formation and germline cell division. Dev. Biol. 365, 384–394. doi: 10.1016/j.ydbio.2012.03.005, PMID: 22445511 PMC3763516

[ref4] Baulcombe D. (2004). RNA silencing in plants. Nature 431, 356–363. doi: 10.1038/nature02874 15372043

[ref5] Bohmert K. Camus I. Bellini C. Bouchez D. Caboche M. Benning C. (1998). AGO1 defines a novel locus of *Arabidopsis* controlling leaf development. EMBO J. 17, 170–180. doi: 10.1093/emboj/17.1.170, PMID: 9427751 PMC1170368

[ref6] Bolger A. M. Lohse M. Usadel B. (2014). Trimmomatic: a flexible trimmer for Illumina sequence data. Bioinformatics 30, 2114–2120. doi: 10.1093/bioinformatics/btu170, PMID: 24695404 PMC4103590

[ref7] Bollmann S. R. Fang Y. Press C. M. Tyler B. M. Grünwald N. J. (2016). Diverse evolutionary trajectories for small RNA biogenesis genes in the oomycete genus *Phytophthora*. Front. Plant Sci. 7:284. doi: 10.3389/fpls.2016.00284, PMID: 27014308 PMC4791657

[ref8] Bollmann S. R. Press C. M. Tyler B. M. Grünwald N. J. (2018). Expansion and divergence of Argonaute genes in the oomycete genus *Phytophthora*. Front. Microbiol. 9:2841. doi: 10.3389/fmicb.2018.02841, PMID: 30555430 PMC6284064

[ref9] Bottin A. Larche L. Villalba F. Gaulin E. Esquerré-Tugayé M.-T. Rickauer M. (1999). Green fluorescent protein (GFP) as gene expression reporter and vital marker for studying development and microbe-plant interaction in the tobacco pathogen *Phytophthora parasitica var. nicotianae*. FEMS Microbiol. Lett. 176, 51–56. doi: 10.1111/j.1574-6968.1999.tb13641.x, PMID: 10917747

[ref10] Bozkurt T. O. Schornack S. Win J. Shindo T. Ilyas M. Oliva R.. (2011). *Phytophthora infestans* effector AVRblb2 prevents secretion of a plant immune protease at the haustorial interface. Proc. Natl. Acad. Sci. U. S. A. 108, 20832–20837. doi: 10.1073/pnas.1112708109, PMID: 22143776 PMC3251060

[ref11] Brennecke J. Aravin A. A. Stark A. Dus M. Kellis M. Sachidanandam R.. (2007). Discrete small RNA-generating loci as master regulators of transposon activity in *Drosophila*. Cell 128, 1089–1103. doi: 10.1016/j.cell.2007.01.043, PMID: 17346786

[ref12] Buker S. M. Iida T. Bühler M. Villén J. Gygi S. P. Nakayama J.. (2007). Two different Argonaute complexes are required for siRNA generation and heterochromatin assembly in fission yeast. Nat. Struct. Mol. Biol. 14, 200–207. doi: 10.1038/nsmb1211, PMID: 17310250

[ref13] Carthew R. W. Sontheimer E. J. (2009). Origins and mechanisms of miRNAs and siRNAs. Cell 136, 642–655. doi: 10.1016/j.cell.2009.01.035, PMID: 19239886 PMC2675692

[ref14] Cervantes M. Vila A. Nicolás F. E. Moxon S. de Haro J. P. Dalmay T.. (2013). A single argonaute gene participates in exogenous and endogenous RNAi and controls cellular functions in the basal fungus *Mucor circinelloides*. PLoS One 8:e69283. doi: 10.1371/journal.pone.0069283, PMID: 23935973 PMC3720535

[ref15] Chen C. Chen H. Zhang Y. Thomas H. R. Frank M. H. He Y.. (2020). TBtools: an integrative toolkit developed for interactive analyses of big biological data. Mol. Plant 13, 1194–1202. doi: 10.1016/j.molp.2020.06.009, PMID: 32585190

[ref16] Chen X. R. Zhang Y. Li H. Y. Zhang Z. H. Sheng G. L. Li Y. P.. (2019). The RXLR effector PcAvh1 is required for full virulence of *Phytophthora capsici*. Mol. Plant-Microbe Interact. 32, 986–1000. doi: 10.1094/MPMI-09-18-0251-R, PMID: 30811314

[ref17] Chong P. A. Vernon R. M. Forman-Kay J. D. (2018). RGG/RG motif regions in RNA binding and phase separation. J. Mol. Biol. 430, 4650–4665. doi: 10.1016/j.jmb.2018.06.014, PMID: 29913160

[ref18] Conesa A. Götz S. García-Gómez J. M. Terol J. Talón M. Robles M. (2005). Blast2GO: a universal tool for annotation, visualization and analysis in functional genomics research. Bioinformatics 21, 3674–3676. doi: 10.1093/bioinformatics/bti610, PMID: 16081474

[ref19] Cui L. Yin W. Dong S. Wang Y. (2012). Analysis of polymorphism and transcription of the effector gene *Avr1b* in *Phytophthora sojae* isolates from China virulent to *Rps1b*. Mol. Plant Pathol. 13, 114–122. doi: 10.1111/j.1364-3703.2011.00733.x, PMID: 21726400 PMC6638858

[ref20] Czech B. Munafò M. Ciabrelli F. Eastwood E. L. Fabry M. H. Kneuss E.. (2018). piRNA-guided genome defense: from biogenesis to silencing. Annu. Rev. Genet. 52, 131–157. doi: 10.1146/annurev-genet-120417-031441, PMID: 30476449 PMC10784713

[ref21] Dong S. Qutob D. Tedman-Jones J. Kuflu K. Wang Y. Tyler B. M.. (2009). The *Phytophthora sojae* avirulence locus *Avr3c* encodes a multi-copy RXLR effector with sequence polymorphisms among pathogen strains. PLoS One 4:e5556. doi: 10.1371/journal.pone.0005556, PMID: 19440541 PMC2678259

[ref22] Durand-Dubief M. Bastin P. (2003). TbAGO1, an argonaute protein required for RNA interference, is involved in mitosis and chromosome segregation in *Trypanosoma brucei*. BMC Biol. 1:2. doi: 10.1186/1741-7007-1-2, PMID: 14670085 PMC317389

[ref23] Durand-Dubief M. Kohl L. Bastin P. (2003). Efficiency and specificity of RNA interference generated by intra- and intermolecular double stranded RNA in *Trypanosoma brucei*. Mol. Biochem. Parasitol. 129, 11–21. doi: 10.1016/s0166-6851(03)00071-9, PMID: 12798502

[ref24] Fahlgren N. Bollmann S. R. Kasschau K. D. Cuperus J. T. Press C. M. Sullivan C. M.. (2013). *Phytophthora* have distinct endogenous small RNA populations that include short interfering and microRNAs. PLoS One 8:e77181. doi: 10.1371/journal.pone.0077181, PMID: 24204767 PMC3804510

[ref25] Fan G. Yang Y. Li T. Lu W. Du Y. Qiang X.. (2018). A *Phytophthora capsici* RXLR effector targets and inhibits a plant PPIase to suppress endoplasmic reticulum-mediated immunity. Mol. Plant 11, 1067–1083. doi: 10.1016/j.molp.2018.05.009, PMID: 29864524

[ref26] Fang Y. Cui L. Gu B. Arredondo F. Tyler B. M. (2017). Efficient genome editing in the oomycete *Phytophthora sojae* using CRISPR/Cas9. Curr. Protoc. Microbiol. 44:21A.1.1-21A.1.26. doi: 10.1002/cpmc.25, PMID: 28166383

[ref27] Finn R. D. Mistry J. Tate J. Coggill P. Heger A. Pollington J. E.. (2010). The Pfam protein families database. Nucleic Acids Res. 38, D211–D222. doi: 10.1093/nar/gkp985, PMID: 19920124 PMC2808889

[ref28] Fry W. (2008). *Phytophthora infestans*: the plant (and R gene) destroyer. Mol. Plant Pathol. 9, 385–402. doi: 10.1111/j.1364-3703.2007.00465.x, PMID: 18705878 PMC6640234

[ref29] Garcia-Silva M. R. Tosar J. P. Frugier M. Pantano S. Bonilla B. Esteban L.. (2010). Cloning, characterization and subcellular localization of a *Trypanosoma cruzi* argonaute protein defining a new subfamily distinctive of trypanosomatids. Gene 466, 26–35. doi: 10.1016/j.gene.2010.06.012, PMID: 20621168

[ref30] Garcia-Silva M. R. Sanguinetti J. Cabrera-Cabrera F. Franzén O. Cayota A. (2014). A particular set of small non-coding RNAs is bound to the distinctive Argonaute protein of *Trypanosoma cruzi*: insights from RNA-interference deficient organisms. Gene 538, 379–384. doi: 10.1016/j.gene.2014.01.023, PMID: 24463018

[ref31] Gijzen M. Ishmael C. Shrestha S. D. (2014). Epigenetic control of effectors in plant pathogens. Front. Plant Sci. 5:638. doi: 10.3389/fpls.2014.00638, PMID: 25429296 PMC4228847

[ref32] Girard A. Sachidanandam R. Hannon G. J. Carmell M. A. (2006). A germline-specific class of small RNAs binds mammalian Piwi proteins. Nature 442, 199–202. doi: 10.1038/nature04917, PMID: 16751776

[ref33] Gleave A. P. (1992). A versatile binary vector system with a T-DNA organisational structure conducive to efficient integration of cloned DNA into the plant genome. Plant Mol. Biol. 20, 1203–1207. doi: 10.1007/BF00028910, PMID: 1463857

[ref34] Grünwald N. J. Goss E. M. Press C. M. (2008). *Phytophthora ramorum:* a pathogen with a remarkably wide host range causing sudden oak death on oaks and ramorum blight on woody ornamentals. Mol. Plant Pathol. 9, 729–740. doi: 10.1111/j.1364-3703.2008.00500.x, PMID: 19019002 PMC6640315

[ref35] Huang V. Li L. C. (2014). Demystifying the nuclear function of Argonaute proteins. RNA Biol. 11, 18–24. doi: 10.4161/rna.27604, PMID: 24384674 PMC3929419

[ref36] Huang G. Liu Z. Gu B. Zhao H. Jia J. Fan G.. (2019a). An RXLR effector secreted by *Phytophthora parasitica* is a virulence factor and triggers cell death in various plants. Mol. Plant Pathol. 20, 356–371. doi: 10.1111/mpp.12760, PMID: 30320960 PMC6637884

[ref37] Huang C. Y. Wang H. Hu P. Hamby R. Jin H. (2019b). Small RNAs: big players in plant-microbe interactions. Cell Host Microbe 26, 173–182. doi: 10.1016/j.chom.2019.07.021, PMID: 31415750

[ref38] Hutvagner G. Simard M. J. (2008). Argonaute proteins: key players in RNA silencing. Nat. Rev. Mol. Cell Biol. 9, 22–32. doi: 10.1038/nrm2321 18073770

[ref39] Jia J. (2017). Diversity of Small RNAs and Their Potential Roles in the Regulation of Gene Expression in Phytophthora Parasitica. Ph.D. dissertation, Yangling, Shaanxi, China: Northwest A&F University.

[ref40] Jia J. Lu W. Zhong C. Zhou R. Xu J. Liu W.. (2017). The 25-26 nt small RNAs in *Phytophthora parasitica* are associated with efficient silencing of homologous endogenous genes. Front. Microbiol. 8:773. doi: 10.3389/fmicb.2017.00773, PMID: 28512457 PMC5411455

[ref41] Judelson H. S. (1997). The genetics and biology of *Phytophthora infestans*: modern approaches to a historical challenge. Fungal Genet. Biol. 22, 65–76. doi: 10.1006/fgbi.1997.1006, PMID: 9367653

[ref42] Katiyar-Agarwal S. Morgan R. Dahlbeck D. Borsani O. Villegas A. Jr. Zhu J. K.. (2006). A pathogen-inducible endogenous siRNA in plant immunity. Proc. Natl. Acad. Sci. U. S. A. 103, 18002–18007. doi: 10.1073/pnas.0608258103, PMID: 17071740 PMC1693862

[ref43] Kim D. Langmead B. Salzberg S. L. (2015). HISAT: a fast spliced aligner with low memory requirements. Nat. Methods 12, 357–360. doi: 10.1038/nmeth.3317, PMID: 25751142 PMC4655817

[ref44] Kobayashi H. Tomari Y. (2016). RISC assembly: coordination between small RNAs and Argonaute proteins. Biochim. Biophys. Acta 1859, 71–81. doi: 10.1016/j.bbagrm.2015.08.007, PMID: 26303205

[ref45] Langmead B. Trapnell C. Pop M. Salzberg S. L. (2009). Ultrafast and memory-efficient alignment of short DNA sequences to the human genome. Genome Biol. 10:R25. doi: 10.1186/gb-2009-10-3-r25, PMID: 19261174 PMC2690996

[ref46] Lee H. C. Li L. Gu W. Xue Z. Crosthwaite S. K. Pertsemlidis A.. (2010). Diverse pathways generate microRNA-like RNAs & Dicer-independent small interfering RNAs in fungi. Mol. Cell 38, 803–814. doi: 10.1016/j.molcel.2010.04.005, PMID: 20417140 PMC2902691

[ref47] Liao Y. Smyth G. K. Shi W. (2014). FeatureCounts: an efficient general purpose program for assigning sequence reads to genomic features. Bioinformatics 30, 923–930. doi: 10.1093/bioinformatics/btt656, PMID: 24227677

[ref510] Lingel A. Simon B. Izaurralde E. Sattler M. (2003). Structure and nucleic-acid binding of the *Drosophila* Argonaute 2 PAZ domain. Nature 426, 465–469. doi: 10.1038/nature02123, PMID: 14615801

[ref48] Liu W. Xie Y. Ma J. Luo X. Nie P. Zuo Z.. (2015). IBS: an illustrator for the presentation and visualization of biological sequences. Bioinformatics 31, 3359–3361. doi: 10.1093/bioinformatics/btv362, PMID: 26069263 PMC4595897

[ref49] Love M. I. Huber W. Anders S. (2014). Moderated estimation of fold change and dispersion for RNA-seq data with DESeq2. Genome Biol. 15:550. doi: 10.1186/s13059-014-0550-8, PMID: 25516281 PMC4302049

[ref50] Makarova K. S. Wolf Y. I. van der Oost J. Koonin E. V. (2009). Prokaryotic homologs of Argonaute proteins are predicted to function as key components of a novel system of defense against mobile genetic elements. Biol. Direct 4:29. doi: 10.1186/1745-6150-4-29, PMID: 19706170 PMC2743648

[ref51] Marasovic M. Zocco M. Halic M. (2013). Argonaute and Triman generate dicer-independent priRNAs and mature siRNAs to initiate heterochromatin formation. Mol. Cell 52, 173–183. doi: 10.1016/j.molcel.2013.08.046, PMID: 24095277

[ref52] Meng Y. Zhang Q. Ding W. Shan W. (2014). *Phytophthora parasitica*: a model oomycete plant pathogen. Mycology 5, 43–51. doi: 10.1080/21501203.2014.917734, PMID: 24999436 PMC4066925

[ref53] Meng Y. Zhang Q. Zhang M. Gu B. Huang G. Wang Q.. (2015). The protein disulfide isomerase 1 of *Phytophthora parasitica* (PpPDI1) is associated with the haustoria-like structures and contributes to plant infection. Front. Plant Sci. 6:632. doi: 10.3389/fpls.2015.00632, PMID: 26347756 PMC4539480

[ref54] Millar A. A. Waterhouse P. M. (2005). Plant and animal microRNAs: similarities and differences. Funct. Integr. Genomics 5, 129–135. doi: 10.1007/s10142-005-0145-2, PMID: 15875226

[ref55] Musiyenko A. Majumdar T. Andrews J. Adams B. Barik S. (2012). PRMT1 methylates the single Argonaute of *Toxoplasma gondii* and is important for the recruitment of Tudor nuclease for target RNA cleavage by antisense guide RNA. Cell. Microbiol. 14, 882–901. doi: 10.1111/j.1462-5822.2012.01763.x, PMID: 22309152 PMC3682492

[ref56] Nguyen Q. Iritani A. Ohkita S. Vu B. V. Yokoya K. Matsubara A.. (2018). A fungal Argonaute interferes with RNA interference. Nucleic Acids Res. 46, 2495–2508. doi: 10.1093/nar/gkx1301, PMID: 29309640 PMC5946944

[ref57] Pais M. Yoshida K. Giannakopoulou A. Pel M. A. Cano L. M. Oliva R. F.. (2018). Gene expression polymorphism underpins evasion of host immunity in an asexual lineage of the Irish potato famine pathogen. BMC Evol. Biol. 18:93. doi: 10.1186/s12862-018-1201-6, PMID: 29973156 PMC6032779

[ref58] Qutob D. Chapman B. P. Gijzen M. (2013). Transgenerational gene silencing causes gain of virulence in a plant pathogen. Nat. Commun. 4:1349. doi: 10.1038/ncomms2354, PMID: 23322037 PMC3562452

[ref59] Qutob D. Tedman-Jones J. Dong S. Kuflu K. Pham H. Wang Y.. (2009). Copy number variation and transcriptional polymorphisms of *Phytophthora sojae* RXLR effector genes *Avr1a* and *Avr3a*. PLoS One 4:e5066. doi: 10.1371/journal.pone.0005066, PMID: 19343173 PMC2661136

[ref60] Rajyaguru P. Parker R. (2012). RGG motif proteins: modulators of mRNA functional states. Cell Cycle 11, 2594–2599. doi: 10.4161/cc.20716, PMID: 22767211 PMC3873214

[ref61] Shan W. Cao M. Leung D. Tyler B. M. (2004). The Avr1b locus of *Phytophthora sojae* encodes an elicitor and a regulator required for avirulence on soybean plants carrying resistance gene *Rps1b*. Mol. Plant-Microbe Interact. 17, 394–403. doi: 10.1094/MPMI.2004.17.4.394, PMID: 15077672

[ref62] Shi H. Chamond N. Djikeng A. Tschudi C. Ullu E. (2009). RNA interference in *Trypanosoma brucei:* role of the N-terminal RGG domain and the polyribosome association of argonaute. J. Biol. Chem. 284, 36511–36520. doi: 10.1074/jbc.M109.073072, PMID: 19880512 PMC2794767

[ref63] Shi H. Ullu E. Tschudi C. (2004). Function of the trypanosome Argonaute 1 protein in RNA interference requires the N-terminal RGG domain and arginine 735 in the Piwi domain. J. Biol. Chem. 279, 49889–49893. doi: 10.1074/jbc.M409280200, PMID: 15383544

[ref511] Song J. J. Smith S. K. Hannon G. J. Joshua-Tor L. (2004). Crystal structure of Argonaute and its implications for RISC slicer activity. Science 305, 1434–1437. doi: 10.1126/science.1102514, PMID: 15284453

[ref64] Swarts D. C. Makarova K. Wang Y. Nakanishi K. Ketting R. F. Koonin E. V.. (2014). The evolutionary journey of Argonaute proteins. Nat. Struct. Mol. Biol. 21, 743–753. doi: 10.1038/nsmb.2879, PMID: 25192263 PMC4691850

[ref65] Thandapani P. O’Connor T. R. Bailey T. L. Richard S. (2013). Defining the RGG/RG motif. Mol. Cell 50, 613–623. doi: 10.1016/j.molcel.2013.05.021, PMID: 23746349

[ref512] Tolia N. H. Joshua-Tor L. (2007). Slicer and the argonautes. Nat. Chem. Biol. 3, 36–43. doi: 10.1038/nchembio848, PMID: 17173028

[ref66] Trapnell C. Roberts A. Goff L. Pertea G. Kim D. Kelley D. R.. (2012). Differential gene and transcript expression analysis of RNA-seq experiments with TopHat and cufflinks. Nat. Protoc. 7, 562–578. doi: 10.1038/nprot.2012.016, PMID: 22383036 PMC3334321

[ref67] Tyler B. M. (2007). *Phytophthora sojae*: root rot pathogen of soybean and model oomycete. Mol. Plant Pathol. 8, 1–8. doi: 10.1111/j.1364-3703.2006.00373.x, PMID: 20507474

[ref68] Vagin V. V. Sigova A. Li C. Seitz H. Gvozdev V. Zamore P. D. (2006). A distinct small RNA pathway silences selfish genetic elements in the germline. Science 313, 320–324. doi: 10.1126/science.1129333, PMID: 16809489

[ref69] van Poppel P. M. Guo J. van de Vondervoort P. J. Jung M. W. Birch P. R. Whisson S. C.. (2008). The *Phytophthora infestans* avirulence gene *Avr4* encodes an RXLR-dEER effector. Mol. Plant-Microbe Interact. 21, 1460–1470. doi: 10.1094/MPMI-21-11-1460, PMID: 18842095

[ref70] Vetukuri R. R. Åsman A. K. Tellgren-Roth C. Jahan S. N. Reimegård J. Fogelqvist J.. (2012). Evidence for small RNAs homologous to effector-encoding genes and transposable elements in the oomycete *Phytophthora infestans*. PLoS One 7:e51399. doi: 10.1371/journal.pone.0051399, PMID: 23272103 PMC3522703

[ref71] Vetukuri R. R. Avrova A. O. Grenville-Briggs L. J. Van West P. Söderbom F. Savenkov E. I.. (2011). Evidence for involvement of dicer-like, Argonaute and histone deacetylase proteins in gene silencing in *Phytophthora infestans*. Mol. Plant Pathol. 12, 772–785. doi: 10.1111/j.1364-3703.2011.00710.x, PMID: 21726377 PMC6640358

[ref72] Wang L. Chen H. Li J. Shu H. Zhang X. Wang Y.. (2020). Effector gene silencing mediated by histone methylation underpins host adaptation in an oomycete plant pathogen. Nucleic Acids Res. 48, 1790–1799. doi: 10.1093/nar/gkz1160, PMID: 31819959 PMC7039004

[ref73] Wang Q. Han C. Ferreira A. O. Yu X. Ye W. Tripathy S.. (2011a). Transcriptional programming and functional interactions within the *Phytophthora sojae* RXLR effector repertoire. Plant Cell 23, 2064–2086. doi: 10.1105/tpc.111.086082, PMID: 21653195 PMC3160037

[ref74] Wang Q. Li T. Zhong C. Luo S. Xu K. Gu B.. (2019). Small RNAs generated by bidirectional transcription mediate silencing of RXLR effector genes in the oomycete *Phytophthora sojae*. Phytopathol. Res. 1:18. doi: 10.1186/s42483-019-0026-6

[ref75] Wang Y. Meng Y. Zhang M. Tong X. Wang Q. Sun Y.. (2011b). Infection of *Arabidopsis thaliana* by *Phytophthora parasitica* and identification of variation in host specificity. Mol. Plant Pathol. 12, 187–201. doi: 10.1111/j.1364-3703.2010.00659.x, PMID: 21199568 PMC6640465

[ref76] Wang Y. Wang Y. (2018). *Phytophthora sojae* effectors orchestrate warfare with host immunity. Curr. Opin. Microbiol. 46, 7–13. doi: 10.1016/j.mib.2018.01.008, PMID: 29454192

[ref77] Wei W. Ba Z. Gao M. Wu Y. Ma Y. Amiard S.. (2012). A role for small RNAs in DNA double-strand break repair. Cell 149, 101–112. doi: 10.1016/j.cell.2012.03.002 22445173

[ref78] Wesley S. V. Helliwell C. A. Smith N. A. Wang M. B. Rouse D. T. Liu Q.. (2001). Construct design for efficient, effective and high-throughput gene silencing in plants. Plant J. 27, 581–590. doi: 10.1046/j.1365-313x.2001.01105.x, PMID: 11576441

[ref79] Xue Z. Yuan H. Guo J. Liu Y. (2012). Reconstitution of an Argonaute-dependent small RNA biogenesis pathway reveals a handover mechanism involving the RNA exosome and the exonuclease QIP. Mol. Cell 46, 299–310. doi: 10.1016/j.molcel.2012.03.019, PMID: 22516970 PMC3351553

[ref80] Ye R. Chen Z. Lian B. Rowley M. J. Xia N. Chai J.. (2016). A dicer-independent route for biogenesis of siRNAs that direct DNA methylation in *Arabidopsis*. Mol. Cell 61, 222–235. doi: 10.1016/j.molcel.2015.11.015, PMID: 26711010 PMC5110219

[ref81] Yigit E. Batista P. J. Bei Y. Pang K. M. Chen C. C. Tolia N. H.. (2006). Analysis of the *C. elegans* Argonaute family reveals that distinct Argonautes act sequentially during RNAi. Cell 127, 747–757. doi: 10.1016/j.cell.2006.09.033, PMID: 17110334

[ref82] Yin W. Xiao Y. Niu M. Meng W. Li L. Zhang X.. (2020). ARGONAUTE2 enhances grain length and salt tolerance by activating BIG GRAIN3 to modulate cytokinin distribution in rice. Plant Cell 32, 2292–2306. doi: 10.1105/tpc.19.00542, PMID: 32409321 PMC7346564

[ref83] Zhang Q. Li W. Yang J. Xu J. Meng Y. Shan W. (2020). Two *Phytophthora parasitica* cysteine protease genes, *PpCys44* and *PpCys45*, trigger cell death in various *Nicotiana* spp. and act as virulence factors. Mol. Plant Pathol. 21, 541–554. doi: 10.1111/mpp.12915, PMID: 32077241 PMC7060141

[ref84] Zheng X. McLellan H. Fraiture M. Liu X. Boevink P. C. Gilroy E. M.. (2014). Functionally redundant RXLR effectors from *Phytophthora infestans* act at different steps to suppress early flg22-triggered immunity. PLoS Pathog. 10:e1004057. doi: 10.1371/journal.ppat.1004057, PMID: 24763622 PMC3999189

