# Supplementary material for: Mutations in PpAGO3 Lead to Enhanced Virulence of Phytophthora parasitica by Activation of 25–26 nt sRNA-Associated Effector Genes
Source: Front Microbiol. 2022 Mar 24;13:856106. doi: 10.3389/fmicb.2022.856106 (PMC8989244; doi:10.3389/fmicb.2022.856106)
Supplement: Supplementary file 1 [file Data_Sheet_1.ZIP › Table S1.docx]

| **Supplementary Table 1.** The sRNA mapping results of *Phytophthora parasitica* in  wild-type and *PpAGO3^ΔRGG1/3^* mutants | | | |
| --- | --- | --- | --- |
| **Sample** | **Raw data** | **Genome mapped reads** | **Candidated reads(18-45nt)** |
| WT1 | 18811564 | 7631715 (40.57%) | 6122342 |
| WT2 | 16958984 | 6991325 (41.22%) | 4811186 |
| WT3 | 16354590 | 6267690 (38.32%) | 4050821 |
| *PpAGO3^ΔRGG1^-1* | 16655603 | 6914399 (41.51%) | 6083448 |
| *PpAGO3^ΔRGG1^-2* | 12922095 | 5728506 (44.33%) | 4978610 |
| *PpAGO3^ΔRGG1^-3* | 12435233 | 5470175 (43.99%) | 4528937 |
| *PpAGO3^ΔRGG3^-1* | 14553641 | 5952660 (40.90%) | 5187448 |
| *PpAGO3^ΔRGG3^-2* | 16774826 | 7104616 (42.35%) | 6107563 |
| *PpAGO3^ΔRGG3^-3* | 14662508 | 5734460 (39.11%) | 5038953 |
