# Supplementary material for: Mutations in PpAGO3 Lead to Enhanced Virulence of Phytophthora parasitica by Activation of 25–26 nt sRNA-Associated Effector Genes
Source: Front Microbiol. 2022 Mar 24;13:856106. doi: 10.3389/fmicb.2022.856106 (PMC8989244; doi:10.3389/fmicb.2022.856106)
Supplement: Supplementary file 1 [file Data_Sheet_1.ZIP › Table S4.docx]

| **Supplementary Table 4.** Primers used in this study | |
| --- | --- |
| **Primer_name** | **Sequence (5' - 3')** |
| PpAGO3-sense (for SgRNA) | CTAGCACCCTCCTGATGAGTCCGTGAGGACGAAACGAGTAAGCTCGTCGAGGGTACGACCAGCGTGGA |
| PpAGO3-antisense (for SgRNA) | AAACTCCACGCTGGTCGTACCCTCGACGAGCTTACTCGTTTCGTCCTCACGGACTCATCAGGAGGGTG |
| PpAGO3-seqF (target site) | AAACCGTCCAGGAAGCG |
| PpAGO3-seqR (target site) | ACGAGCCACCGTAACCAG |
| PPTG_01869_GFPF | ACGCTCGAGGAATTCGGTACCATGGTGAGCAAGGGCGAGG |
| PPTG_01869_GFPR | GCGGATATCTTGTACAGCTCGTCCATGCC |
| PPTG_01869_F | GAGCTGTACAAGATATCCGCTTTTGTCACTGAACAA |
| PPTG_01869_R | TCATTAAAGCAGGACTCTAGATCACAAGGCGCTAACCGC |
| PPTG_15425_GFPF | ACGCTCGAGGAATTCGGTACCATGGTGAGCAAGGGCGAGG |
| PPTG_15425_GFPR | TCAAGACCTTGTACAGCTCGTCCATGCC |
| PPTG_15425_F | CGAGCTGTACAAGGTCTTGAAATCAACGGTGCTGC |
| PPTG_15425_R | TCATTAAAGCAGGACTCTAGATTAGTTCAAATTACTAGGCGATGTT |
