# Supplementary material for: Mutations in PpAGO3 Lead to Enhanced Virulence of Phytophthora parasitica by Activation of 25–26 nt sRNA-Associated Effector Genes
Source: Front Microbiol. 2022 Mar 24;13:856106. doi: 10.3389/fmicb.2022.856106 (PMC8989244; doi:10.3389/fmicb.2022.856106)
Supplement: Supplementary file 1 [file Data_Sheet_1.ZIP › Supplementary Figure 1.pdf]

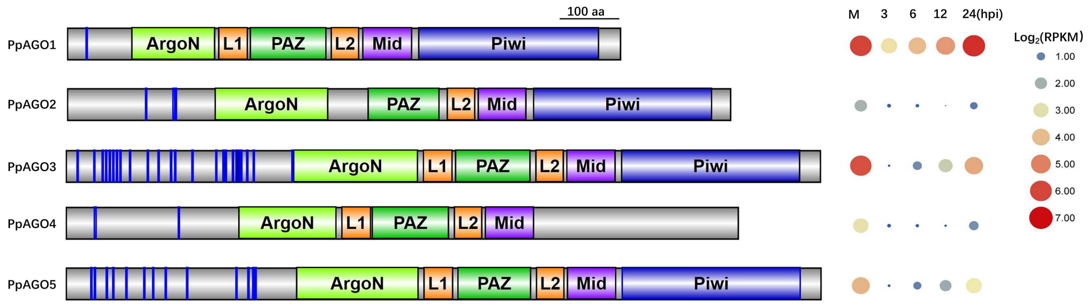

Supplementary Figure 1. Bioinformatic analysis of AGO proteins in *P. parasitica*. (left) The five predicted PpAGO proteins were conserved and contain the following typical domains: ArgoN, amino-terminal; L1, Linker 1; PAZ, Piwi-Argonaute-Zwille; L2, Linker 2; Mid, ArgoMid; Piwi, Piwi-Argonaute-Zwille. Besides that, all five PpAGO proteins contain the RGG (argine-glycine-glycine) domain, marked by blue color lines. PpAGO proteins were identified by using PiAGO amino acid sequences to BLASTP against *P. parasitica* protein database and domain architectures were predicted by using Pfam database. These PpAGO proteins include PPTG_07447 (XP_008899978) (PpAGO1), PPTG_12053 (XP_008906498) (PpAGO2), PPTG_12013 (XP_008906444) (PpAGO3), PPTG_12064 (XP_008906511) (PpAGO4), and PPTG_23076 (XP_008906512) (PpAGO5). The expression of *PpAGO1*, *PpAGO3* and *PpAGO5* was highly upregulated in the mycelium stage but sharply reduced at the early infection stages. For *PpAGO2* and *PpAGO4*, the gene expression level was relatively low in mycelium and even could not be detected during infection stages. The gene expression pattern was obtained by analyzing RNA-seq data conducted by Jia (2017) in which the 6-week old *N. benthamiana* detached leaves at 3, 6, 12, 24 hour post inoculated (hpi) with *P. parasitica* zoospores. The circle size indicates gene expression level, according to the transformation value of log_2(FPKM), (FPKM=Fragment Per Kilobase per Million mapped reads). M, mycelium stage.
