# Supplementary material for: Mutations in PpAGO3 Lead to Enhanced Virulence of Phytophthora parasitica by Activation of 25–26 nt sRNA-Associated Effector Genes
Source: Front Microbiol. 2022 Mar 24;13:856106. doi: 10.3389/fmicb.2022.856106 (PMC8989244; doi:10.3389/fmicb.2022.856106)
Supplement: Supplementary file 1 [file Data_Sheet_1.ZIP › Supplementary Figure 2.pdf]

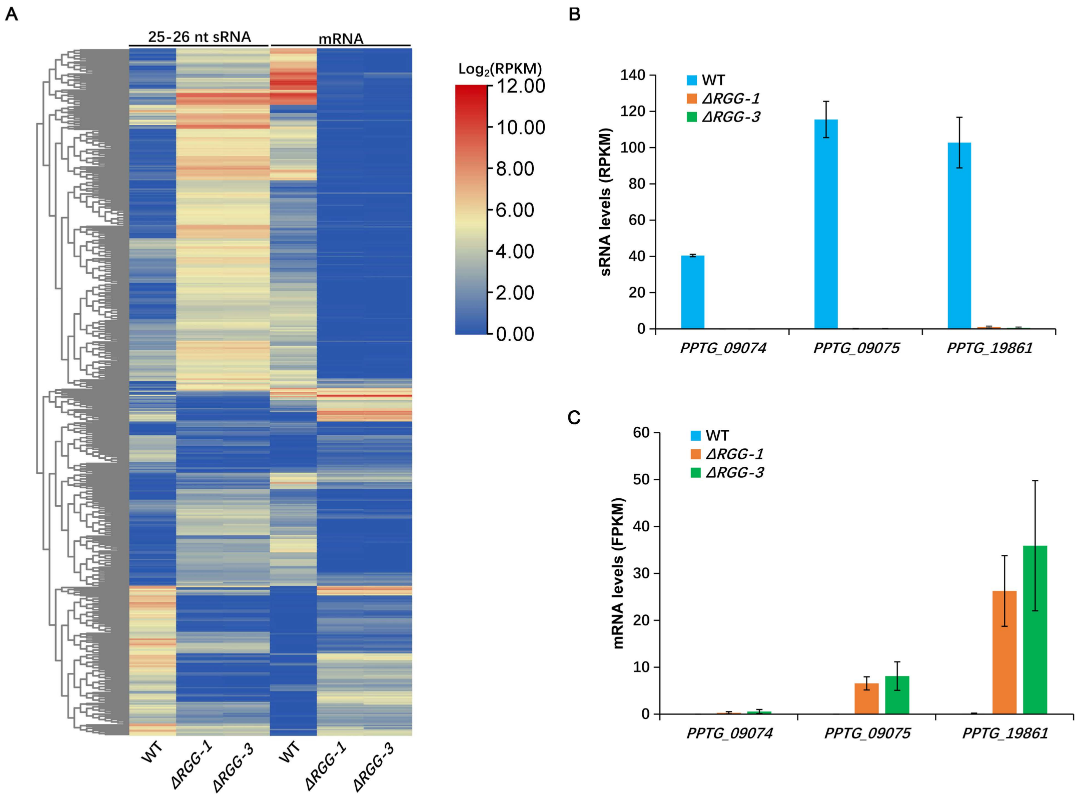

Supplementary Figure 2. The changed accumulation of 25-26 nt sRNAs and associated transcripts in mutants *PpAGO3*$^{\Delta RGG1}$ and *PpAGO3*$^{\Delta RGG3}$ is negatively correlated. **(A)** Heatmap for the expression of 25-26 nt sRNAs and associated transcripts of 509 non-cytoplasmic effector genes in the wild-type, and mutants *PpAGO3*$^{\Delta RGG1}$ and *PpAGO3*$^{\Delta RGG3}$. **(B-C)** The histogram represents expression of 25-26 nt sRNAs and associated transcripts of three INF-like genes (*PPTG_09074*, *PPTG_09075* and *PPTG_19861*).
